# Clinical effects of teriparatide, abaloparatide, and romosozumab in postmenopausal osteoporosis

**DOI:** 10.1007/s00774-024-01536-0

**Published:** 2024-07-15

**Authors:** Kosuke Ebina, Yuki Etani, Takaaki Noguchi, Ken Nakata, Seiji Okada

**Affiliations:** 1 Department of Orthopaedic Surgery, Osaka University Graduate School of Medicine, 2-2 Yamada-Oka, Suita, Osaka 565-0871 Japan; 2 Department of Sports Medical Biomechanics, Osaka University Graduate School of Medicine, 2-2 Yamada-Oka, Suita, Osaka 565-0871 Japan; 3 Department of Health and Sport Sciences, Osaka University Graduate School of Medicine, 2-2 Yamada-Oka, Suita, Osaka 565-0871 Japan

**Keywords:** Abaloparatide, Osteoanabolic agent, Postmenopausal osteoporosis, Romosozumab, Teriparatide

## Abstract

In the management of osteoporosis, anti-resorptive agents serve as a primary therapeutic approach. However, in cases where individuals exhibit an increased susceptibility to fractures, such as those characterized by severe low bone mass or a history of vertebral or hip fractures that markedly diminish life expectancy, the immediate reduction of fracture risk through the administration of osteoanabolic agents could be beneficial. Teriparatide, available in daily, once-weekly, or twice-weekly dosages, along with abaloparatide and romosozumab, constitutes a trio of such agents. Each of these medications is defined by unique characteristics, distinct efficacy profiles, and specific adverse effects. There is growing evidence to suggest that these agents have a superior effect on enhancing bone mineral density and reducing fracture incidence when compared to traditional bisphosphonate therapies. Nonetheless, their employment demands thorough consideration of clinical indications, which includes evaluating economic factors, the frequency of injections required, and the potential for adverse effects. The objective of this review is to consolidate the current evidence focusing primarily on the efficacy of these agents, with the goal of enhancing understanding and aiding in making more informed treatment decisions, particularly for those individuals who are at an elevated risk of fractures.

## Introduction

The primary objective in osteoporosis management is fracture prevention through the enhancement of bone mass and strength [1]. Anti-resorptive drugs, which target osteoclast inhibition to reduce bone resorption, have shown efficacy in reducing fracture risk and increasing bone mineral density (BMD), albeit with a concomitant reduction in bone formation [2]. However, their prolonged use has been linked to adverse effects such as atypical femoral fractures and osteonecrosis of the jaw [3]. Although the relative risk of these adverse events is generally low, the severity of skeletal effects has prompted concerns regarding their prolonged usage.

Recent studies have underscored the strong correlation between on-treatment BMD gain and reduced fracture risk, emphasizing the efficacy of BMD improvement in fracture prevention [4]. The International Osteoporosis Foundation has issued guidelines defining treatment failure in osteoporosis, suggesting consideration of treatment change for patients experiencing significant BMD decreases, such as 5% or more at the lumbar spine (LS) or 4% at the proximal femur [5]. This recommendation includes the substitution of oral drugs with injected ones and the replacement of strong anti-resorptives with osteoanabolic agents.

To stimulate new bone formation and achieve greater BMD increases, osteoanabolic agents such as teriparatide, abaloparatide, and romosozumab have been developed (Table 1) [2, 6]. Teriparatide and abaloparatide act as parathyroid hormone (PTH) receptor 1 agonists, primarily stimulating remodeling-based bone formation, while romosozumab primarily induces modeling-based bone formation [7]. Head-to-head clinical trials have demonstrated the superiority of osteoanabolic agents over bisphosphonates in reducing fracture risk, particularly in high-risk patients [8]. Their enhanced efficacy has generated high expectations regarding their primary role in severe osteoporosis treatment.

**Table 1:** Summary of approved osteoanabolic agents

Drugs	Teriparatide	Abaloparatide	Romosozumab
Molecule	PTH (1–34)	PTHrP (1–34) analog	Humanized IgG2 monoclonal antibody
Mechanism	PTH1 receptor agonist	PTH1 receptor agonist	Anti-sclerostin
Dose	20 µg SC daily, 28.2 µg SC twice weekly, 56.5 µg SC weekly	80 µg SC daily	210 mg SC monthly
Approval	Daily (2002), weekly (2011 Japan, 2016 Korea), twice weekly (2019 Japan)	2017	2019
Administration	Daily, twice weekly (Self-injection), weekly (By healthcare professional)	Self-injection	By healthcare professional
Duration limit	24 months	24 months lifetime (18 months in Japan)	12 months (may repeat)
Bone formation	Daily (increase), twice weekly (increase), weekly (increase)	Increase	Increase
Bone resorption	Daily (increase), twice weekly (decrease), weekly (decrease)	Increase	Decrease

*PTH* parathyroid hormone, *PTHrP* parathyroid hormone-related peptide, *SC* subcutaneous injection

This review provides an in-depth analysis of the clinical data on these osteoanabolic therapies, summarizing efficacy information and aiding in clinical decision-making regarding their use in osteoporosis management.

## Osteoanabolic agents

PTH comprises 84 amino acids and activates PTH1 receptors in kidney and bone to regulate calcium metabolism [9]. Teriparatide is a synthetic peptide generated using recombinant DNA technology, consisting of the first 34 amino acids of PTH [9]. Intermittent exposure to PTH directly stimulates osteoblasts and osteocytes in an osteoanabolic manner, promotes stem cell differentiation into osteoblasts, and enhances the activity of osteoblasts and extends their life span [9]. Furthermore, PTH decreases sclerostin expression, a bone formation inhibitor primarily produced by osteocytes, further promoting bone formation [10]. Conversely, PTH stimulates the production of receptor activator of nuclear factor kappa-β ligand (RANKL), enhancing the activity of osteoclast and bone resorption.

Moreover, once-weekly (56.5 µg; administered by healthcare professional; approved in 2011 in Japan and in 2016 in Korea) [11] and twice-weekly (28.2 µg; self-injection; approved in 2019 in Japan) [12] formulations of teriparatide have been developed and are widely used in Japan (Table 1).

Abaloparatide, a synthetic analog of parathyroid hormone-related peptide (PTHrP), consists of 34 amino acids and shares significant homology with human PTHrP 1–34 and teriparatide [9]. Abaloparatide activates the PTH1 receptor on osteoblasts and osteocytes similar to teriparatide. Compared to teriparatide, abaloparatide demonstrates selective preference for the G protein-dependent (GTPγS-sensitive) receptor (RG) conformation of the PTH receptor, leading to reduced duration of intracellular activity, reduced calcemic response, and RANKL production [13]. Both teriparatide and abaloparatide enhance osteoblast and osteoclast activity, primarily at active remodeling sites, facilitating the creation of new bone formation spaces [9].

Romosozumab is a humanized IgG2 monoclonal antibody against sclerostin, a glycoprotein mainly derived from osteocytes that inhibits bone formation through its interaction with low-density lipoprotein receptor-related proteins 5 and 6, thereby inhibiting canonical Wnt signaling in bone [6]. By antagonizing sclerostin, romosozumab promotes bone formation and concurrently decreases the expression of RANKL, thereby reducing bone resorption, resulting in a "dual effect". Consequently, the bone formation increase induced by romosozumab primarily involves modeling-based mechanisms without bone resorption, leading to rapid gains in bone mass [9]. A summary of these osteoanabolic agents is provided in Table 1.

## Efficacy

### Teriparatide

**Fig. 1:**
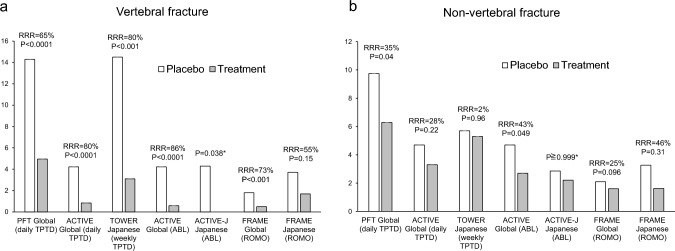
Incidence of **a** vertebral and **b** non-vertebral fractures in treatment versus placebo groups in pivotal trials of osteoanabolic agents among women with postmenopausal osteoporosis. Bar heights indicate the fracture incidence in both placebo and treatment groups. Each treatment group's relative risk reduction, with corresponding P value, is provided. Additionally, the studies' names, countries of the patient populations, and names of the agents are detailed. Note: Due to variations in patient populations and follow-up durations across the studies, comparisons between studies are not recommended. *TPTD* teriparatide, *ABL* abaloparatide, *ROMO* romosozumab. *PFT* pivotal fracture trial [14]; *ACTIVE* Abaloparatide Comparator Trial in Vertebral Endpoints Trial [17], ACTIVE-J [18]; *TOWER* teriparatide once-weekly efficacy research [11], *FRAME* Fracture Study in Postmenopausal Women with Osteoporosis study [21], FRAME Japanese [EVENITY (Common Technical Document, M2.7.3). Tokyo, Japan: Amgen KK, Inc.; 2019]. *RRR* relative risk reduction. *RRR not provided

In the phase III Pivotal Fracture Trial (PFT), involving a high fracture risk cohort of 1,637 postmenopausal women with previous vertebral fractures, daily administration of 20-μg teriparatide yielded a remarkable 65% reduction in the relative risk of vertebral fractures and a 35% reduction in non-vertebral fractures compared to placebo (Fig. 1a and b) [14]. Additionally, this study demonstrated concurrent increases in BMD from baseline, with an 8.6% rise in the lumbar spine (LS) and a 3.6% increase in total hip (TH) compared to placebo (Table 2) [14]. Although the 40-μg dose of teriparatide showed greater increases in BMD and a reduction in fracture frequency compared to the 20-μg dose, it was associated with higher rates of adverse events such as hypercalcemia, dizziness, and nausea, leading to the approval of only the 20-μg dose for postmenopausal osteoporosis treatment [14].

**Table 2:** BMD changes associated with each osteoanabolic agent compared to placebo

Osteoanabolic agent	Study name [references]	Treatment interval	Mean % BMD difference from placebo		
			LS	TH	FN
Teriparatide (20 µg daily)	PFT [14]	18 months	8.6%^***^	3.6%^***^	3.5%^***^
Teriparatide (56.5 µg weekly)	TOWER [11] (Japanese)	18 months	6.4%^**^	3.0%^**^	2.3% ^N.A^
Teriparatide (28.2 µg twice weekly)	TWICE [12] (Japanese)	12 months	1.3% (vs. weekly)^**^	N.A	N.A
Abaloparatide (80 µg daily)	ACTIVE [17]	18 months	10.4%^***^	4.3%^***^	4.0%^***^
	ACTIVE-J [18] (Japanese)	18 months	12.5%^***^	4.3%^***^	4.3%^***^
Romosozumab (210 mg monthly)	FRAME [21]	12 months	13.3%^***^	6.9%^***^	5.9%^***^
	FRAME [CTD] (Japanese)	12 months	14.9%^***^	4.8%^***^	4.6%^***^

**P < 0.01, ***P < 0.001 vs. placebo

*BMD* bone mineral density, *LS* lumbar spine, *TH* total hip, *FN* femoral neck, *N.A*. not applicable, *CTD EVENITY* Common Technical Document, M2.7.3. Tokyo, Japan: Amgen KK, Inc.; 2019

Following the discontinuation of treatment in the PFT, 1262 women participated in a subsequent follow-up study. Over a median period of 30 months post-teriparatide discontinuation, the risk of radiographic vertebral fractures was reduced by 41%, and the risk of non-vertebral fractures was lowered by 36% in the groups treated with 20-μg of teriparatide compared to those receiving placebo, indicating sustained anti-fracture efficacy after teriparatide discontinuation [15].

In the VERtebral fracture treatment comparisons in Osteoporotic women (VERO) study, teriparatide was evaluated against risedronate in postmenopausal women with low BMD and a history of fractures. The group receiving teriparatide demonstrated significantly reduced incidences of new vertebral fractures (5.4% compared to 12%) and clinical fractures (4.8% compared to 9.8%) relative to the risedronate group [16].

The efficacy of once-weekly 56.5-µg teriparatide was evaluated in Japanese patients with primary osteoporosis and a high risk of fracture in the Teriparatide Once-Weekly Efficacy Research (TOWER) trial. In this trial, once-weekly administration of teriparatide significantly reduced the risk of new vertebral fractures, with an incidence of 3.1% in the teriparatide group compared to 14.5% in the placebo group (*P* < 0.01) as shown in Fig. 1a. Furthermore, at 72 weeks, there was a significant increase in BMD in the teriparatide group, with gains of 6.4% at the LS, 3.0% at the TH, and 2.3% at the femoral neck (FN) compared to placebo (*P* < 0.01), as detailed in Table 2 [11].

In the TWICE study, a 48-week, randomized non-inferiority trial investigated the efficacy and safety of 28.2-µg twice-weekly versus 56.5-µg once-weekly teriparatide regimens. At the final follow-up, the LS BMD increased by 7.3% in the 28.2-µg twice-weekly group and 5.9% in the 56.5-µg once-weekly group (*P* < 0.01), as reported in Table 2. There were no significant differences in the increases in BMD at the TH and FN between the two groups. Furthermore, adverse events such as nausea, vomiting, and pyrexia were significantly less frequent in the 28.2-µg twice-weekly group compared to the 56.5-µg once-weekly group (39.7% vs. 56.2%; *P* < 0.01) [12].

## Abaloparatide

In the Abaloparatide Comparator Trial in Vertebral Endpoints (ACTIVE), a phase III clinical trial, the efficacy of abaloparatide was assessed in postmenopausal women with osteoporosis and a high risk of fractures over 18 months, comparing its effects to those of placebo and teriparatide [17]. Treatment with abaloparatide led to statistically significant increase in BMD at the LS, TH, and FN compared to placebo [17]. While changes in LS BMD were comparable between abaloparatide and teriparatide, abaloparatide showed superior outcomes at the TH and FN [17]. Both abaloparatide and teriparatide significantly reduced the risk of vertebral fractures compared to placebo, with reductions of 86% and 80%, respectively (Fig. 1a) [17]. Although abaloparatide demonstrated a lower incidence of major osteoporotic fractures compared to teriparatide (1.5% vs. 3.1%), no statistically significant differences were found in the rates of non-vertebral fractures, with abaloparatide and teriparatide showing reductions of 43% and 28% compared to placebo, respectively, as depicted in Fig. 1b [17]. Similar efficacy was shown in Japanese patients of the ACTIVE study population [18].

Prospective responder analyses of abaloparatide trials underscored its efficacy, with a significantly greater number of patients achieving increases in BMD of > 3% and > 6% at all evaluated sites (LS, TH, and FN) across various time points, compared to both placebo and teriparatide (*P* < 0.001 for all comparisons between abaloparatide and placebo, and abaloparatide and teriparatide) [19].

Additionally, results from the ACTIVE trial suggested that abaloparatide may demonstrate superior efficacy compared to teriparatide, as evidenced by a lower number needed to treat (NNT) to prevent one vertebral or non-vertebral, clinical, or major osteoporotic fracture [20].

## Romosozumab

In the Fracture Study in Postmenopausal Women with Osteoporosis (FRAME) trial, romosozumab demonstrated a 73% reduction in the risk of new vertebral fractures over 12 months compared to placebo in postmenopausal women (Fig. 1a) [21]. Although there were reductions in non-vertebral and clinical fracture risks, these did not achieve statistical significance, with a relative risk reduction of 25% (*P* = 0.096) (Fig. 1b) [21].

In the Active-Controlled Fracture Study in Postmenopausal Women with Osteoporosis at High Risk (ARCH) clinical trial, romosozumab was compared with alendronate over one year. Romosozumab significantly enhanced BMD gains over alendronate after the initial 12 months [22]. Furthermore, romosozumab showed superiority in reducing risks of new vertebral, clinical, non-vertebral, and hip fractures by 48%, 27%, 19%, and 38%, respectively [22]. Although the risks of atypical femoral fractures and osteonecrosis of the jaw were comparable between the two groups, romosozumab was associated with more frequent serious cardiovascular events compared to alendronate [22].

Tomographic and histomorphometric analysis of patients from the FRAME trial indicated an early, albeit transient, increase in bone formation at 2 months post-treatment, followed by sustained suppression of bone resorption up to 12 months [23]. These changes suggest a phase of intense bone modeling, independent of increased resorption or coupled remodeling.

The Study to Evaluate the Effect of Treatment With Romosozumab Compared with Teriparatide in Postmenopausal Women at High Risk of Fracture Previously Treated with a Bisphosphonate (STRUCTURE) demonstrated that romosozumab led to greater BMD increases at the LS and TH than teriparatide after 12 months [24]. Additionally, romosozumab resulted in more significant changes in estimated bone strength at both skeletal sites [25, 26].

A network meta-analysis revealed that teriparatide, abaloparatide, and romosozumab significantly reduced the relative risk of vertebral and non-vertebral fractures compared to placebo, with romosozumab uniquely showing a significant reduction in hip fractures compared to placebo (Table 3) [27]. However, recent observational studies have suggested that prior treatment with anti-resorptive agents, such as bisphosphonates and denosumab, may attenuate the increase in BMD induced by romosozumab [28, 29], even within 12 months of treatment [30].

**Table 3:** Network meta-analysis of the fracture risk with each osteoanabolic agent (reference [27])

Fracture site	Comparator	Teriparatide	Abaloparatide	Romosozumab
Vertebral	Placebo	0.27 (0.19–0.38)^*^	0.14 (0.05–0.42)^*^	0.33 (0.22–0.49)^*^
Non-vertebral	Placebo	0.62 (0.47–0.80)^*^	0.51 (0.29–0.87)^*^	0.67 (0.53–0.86)^*^
Hip	Placebo	0.64 (0.25–1.68)	0.24 (0.01–4.84)	0.44 (0.24–0.79)^*^

Data are expressed as relative risks (95% confidence interval)

*Statistically significant

## Conclusions

Anti-resorptive agents are the foundational treatment for established osteoporosis, acknowledged for their well-established efficacy [31]. Nonetheless, postmenopausal women are at the highest risk of subsequent fractures within the first year following an initial fracture [32]. Osteoanabolic drugs have demonstrated a quicker and more effective reduction in fracture risk compared to oral bisphosphonates. For patients at very high fracture risk—such as postmenopausal women with a T-score below −2.5 and with two or more vertebral or hip fractures, which significantly impact life expectancy—initiating treatment with an osteoanabolic agent over an anti-resorptive agent may be more appropriate [33].

Despite the proven efficacy of osteoanabolic therapies, their use is limited by factors such as high costs, the requirement for routine injections, and potential adverse effects. Therefore, when choosing a therapeutic approach, careful consideration of the patient’s immediate fracture risk and the strategy for sequential therapy is essential. In conclusion, understanding the mechanisms of action and effectiveness of osteoanabolic agents can enable more personalized management of osteoporosis in patients with a high risk of fractures.
